# Supplementary material for: Endogenous RNAi pathway evolutionarily shapes the destiny of the antisense lncRNAs transcriptome
Source: Life Sci Alliance. 2019 Aug 28;2(5):e201900407. doi: 10.26508/lsa.201900407 (PMC6713810; doi:10.26508/lsa.201900407)
Supplement: Supplementary file 3 [file LSA-2019-00407_TableS3.docx]

**Table S3**. ***S*trains.**

| **Strain ID** | **Genotype** | **Source/Reference** |
| --- | --- | --- |
| YAM2478 | *MATα ura3-1 hoΔ* | DBP005 (Drinnenberg et al, 2009) |
| YAM2479 | *MATα ura3-1 hoΔ xrn1Δ::kanMX6* | This work |
| YAM2795 | *MATα ura3-1 hoΔ dcr1Δ* | DBP318 (Drinnenberg et al, 2009) |
| YAM2796 | *MATα ura3-1 hoΔ dcr1Δ xrn1Δ::kanMX6* | This work |
| YAM2826 | *MATα ura3-1 hoΔ dcr1-GFP(S65T)-kanMX6* | This work |
| YAM2842 | *MATα ura3-1 hoΔ dcr1Δ::GFP(S65T)-kanMX6* | This work |
