# Supplementary material for: Endogenous RNAi pathway evolutionarily shapes the destiny of the antisense lncRNAs transcriptome
Source: Life Sci Alliance. 2019 Aug 28;2(5):e201900407. doi: 10.26508/lsa.201900407 (PMC6713810; doi:10.26508/lsa.201900407)
Supplement: Supplementary file 4 [file LSA-2019-00407_TableS4.docx]

**Table S4**. **Oligonucleotides.**

| **ID** | **Sequence 5’-3’** | **Target** | **Use** |
| --- | --- | --- | --- |
| AMO1964 | GGGGTACCAAAAATTGAAAAATTCTGGGC | *XRN1* | PCR (Cloning) |
| AMO1965 | CGGGATCCGATTAAAATGAATGTAAATTTATGTTACA | *XRN1* | PCR (Cloning) |
| AMO1966 | CGGAATTCGAGTATCCGTTGAATGACATTTAAA | *XRN1* | PCR (Cloning) |
| AMO1967 | GCTCTAGATATTGATTTGAGAGAAGAAGCG | *XRN1* | PCR (Cloning) |
| AMO1996 | CAGTACTACTCAATTGCTCTCGAGC | *XRN1* | PCR |
| AMO1997 | GTTGAAGAAAGAGCAGGAACTCTCC | *XRN1* | PCR |
| AMO1998 | TCTTGGTTACATCGTCGTCGTTACC | *XRN1* | Northern-blot |
| AMO2000 | GAAACGTGCAATCCATGTCTGACCG | *scR1* | Northern-blot |
| AMO2001 | CCAGAAGGAAAGGCCCGGTTGGA | 18S rRNA | Northern-blot |
| AMO2002 | AAATTTAATAATTGGGTCGAATCGTAAGGG | 5’ ITS1 | PCR (probe) |
| AMO2003 | TTTGTATTTCATAACGAAATTGGTTTTGAC | 5’ ITS1 | PCR (probe) |
| AMO3227 | TGTAAAATCCTACATTTAAATAGTGC | *XRN1* | PCR |
| AMO3228 | GTCGTAAACTTACAGTTGATGAGG | *XRN1* | PCR |
| AMO3229 | ATGAGTGTTCGAGGTTTAATTAGCG | *DCR1* | PCR |
| AMO3230 | TACGATATCCTAAGTACAGATGCC | *DCR1* | PCR |
| AMO3323 | CGGGGTACCGCCGGCTGTTTCAAATGCACTTGG | *DCR1* | PCR (Cloning) |
| AMO3324 | GCTCTAGAGCCGGCATTCTATAAAGAAAATACTGATGAG | *DCR1* | PCR (Cloning) |
| AMO3325 | AAATATCATTTGAATTCAAAGCTTTGGATCCCCAGATTGTTGCAATGCCTCAAGTATTCC | *DCR1* | PCR (Cloning) |
| AMO3326 | CAACAATCTGGGGATCCAAAGCTTTGAATTCAAATGATATTTATGCACCTTTTATTTATC | *DCR1* | PCR (Cloning) |
| AMO3327 | TACGCTGCAGGTCGACGGATCC | *GFP(S65T)-kanMX6* | PCR (Cloning) |
| AMO3328 | TGGATCTGATATCATCGATGAATTCGAGC | *GFP(S65T)-kanMX6* | PCR (Cloning) |
| AMO3370 | CGGGGTACCGCCGGCTTTAAAACCATGGAATAGACATAG | *DCR1* | PCR (Cloning) |
| AMO3371 | CGCGGATCCCCATTTGTATAATTGCGTGTAGGTCAC | *DCR1* | PCR (Cloning) |
